# The Organisational Drivers That Enabled Health Promoting Policy Change Within Australian Universities—A 2016 Move to Smoke‐Free Campuses Case Study

**DOI:** 10.1002/hpja.70254

**Published:** 2026-10-20

**Authors:** Patricia Taylor, Fiona H. McKay, Catherine M. Bennett

**Affiliations:** ^1^ School of Health and Social Development, Health, Institute of Health Transformation Deakin University Waurn Ponds Australia; ^2^ School of Health and Social Development and the Institute for Health Transformation Deakin University Waurn Ponds Victoria Australia; ^3^ School of Health and Social Development, Institute of Health Transformation Deakin University Waurn Ponds Australia

**Keywords:** health promoting universities, organisational change, policy, settings‐based health promotion, tobacco harm reduction

## Abstract

**Issue Addressed:**

Universities are complex organisational settings for health promotion, operating as workplaces, educational institutions and influential community leaders. In 2016, Australian universities were progressively adopting smoke‐free campus policies within a broader expansion of smoke‐free public policy and tobacco control measures. However, across the sector, implementation was inconsistent. This study examines factors that enabled smoke‐free policy change during this formative period, using university tobacco policy as a case study of health‐promoting organisational change.

**Methods:**

This study conducted semi‐structured interviews in 2016 to explore the experiences of 32 staff across 10 Australian universities and examine organisational and contextual influences on the development and implementation of smoke‐free policies. Data were analysed using an inductive approach informed by Kotter's stages of change.

**Results:**

Similar organisational dynamics supported smoke‐free policy change across institutions. Senior executive support acted as a catalyst for change, while internal champions sustained momentum and operationalised policy implementation. Resource investment, organisational priorities and external influences also shaped implementation.

**Conclusion:**

The findings provide insight into how Australian universities navigated smoke‐free policy change during a formative period of tobacco‐control implementation. While the specific policy context has since evolved, the findings highlight organisational processes that influenced the development and implementation of health promoting policy.

## 1 Introduction

Universities serve as workplaces, educational and research institutions, and as societal leaders. In Australia, universities operate under both national and state legislation that requires the provision of safe environments [1]. Through the strength of their internal policies and practices, and their role in generating research, providing education and influencing public discourse, universities are in a prime position to influence the adoption of health promoting strategies more broadly. Universities, as educators and knowledge producers, can, and should, model and advocate for health‐promoting principles within and beyond campus boundaries [2, 3, 4, 5].

Within Australia, the 2025 Australian Universities Accord requires that the health and wellbeing of university communities be prioritised by institutions through stronger governance and policy [6]. In 2015, universities across the world collaborated to create the Okanagan Charter: An International Charter for Health Promoting Universities and Colleges [3]. Additionally, in 2025, the Limerick Framework for Action was launched [5], which set out 10 transformative actions to empower universities to deliver on the Charter's vision, integrating a global vision with local adaptability, encouraging institutions to shift focus from individual behaviour towards the interconnected roles of planet, place and people. The recommended actions prioritise resourcing, promote collaborative leadership, strengthen partnerships, advance inclusion and empowerment, extend health promotion beyond campus boundaries and embed data‐driven accountability [5]. These frameworks, therefore, support universities in investing in the health of their communities, providing vision and guidance on enabling actions to establish whole‐of‐university health promotion strategies.

Australian universities have increasingly engaged in health promotion across multiple domains, including sexual health, equity and access, disability and wellbeing [7]. However, university health promotion has largely remained focused on individual behaviour change and downstream approaches rather than broader organisational and environmental change [4, 8, 9, 10]. In 2016, this issue was particularly pertinent to tobacco control. Australia had a well‐established national tobacco‐control framework, including comprehensive tobacco advertising restrictions, tobacco taxation, plain packaging and graphic health warnings, alongside progressively strengthened smoke‐free legislation and the National Tobacco Strategy 2012–2018 [11]. By this time, smoke‐free policies were also extending beyond enclosed public places to outdoor environments, including university campuses. Yet implementation across Australian universities remained uneven, with 23 of 43 universities having adopted smoke‐free policies in 2016.

Universities therefore represented an important organisational setting within which broader tobacco‐control objectives were translated into institutional policy and practice. Understanding how this policy change was enabled is particularly relevant given the organisational complexity of universities and the influence of institutional leadership, internal stakeholders and wider social and policy contexts on health‐promoting change. This study examines factors that enabled the development and implementation of smoke‐free tobacco policies in Australian universities during this formative period of policy change.

## 2 Methods

The analysis is informed by Kotter's stages of change [12], being particularly suitable for health promotion within complex settings [12, 13, 14, 15]. These stages of change are categorised as readiness, buy‐in, capacity, engagement and sustainability. The analysis was informed by Kotter's stages of change [12] used during the development of the study protocol and analysis to support an understanding of how health promotion can be developed and implemented within complex organisational settings. This approach is consistent with broader settings‐based health promotion principles articulated through the Okanagan Charter [3] and the Healthy Universities concept, which emphasise whole‐of‐institution approaches to organisational change. The Limerick Framework for Action [5] was not used to inform the original study design or analysis; rather, it was applied retrospectively to situate and interpret the findings within contemporary health‐promoting campus literature and practice. This distinction is important given the data were collected in 2016, prior to the development of the Limerick Framework [5].

### 2.1 Sample and Recruitment

Qualitative methods were employed to gain an in‐depth understanding of staff experience and perspectives on the development and implementation of a smoke‐free policy at 10 Australian universities. The university sample was purposefully chosen in 2016 to incorporate a breadth of institutions based on institution size and location (regional and metropolitan). Universities in the sample were restricted to 23 universities that had a smoke‐free policy in place at that time [16]. An invitation to participate was sent to university Vice Chancellor (VC) offices, followed by individual invitations sent to those identified as having played a key role in smoke‐free policy development and implementation. Further snowball sampling was undertaken with interviewee input to ensure all relevant staff were approached [17]. Participants included senior executives (including VCs), academics and health, wellbeing and/or occupational health and safety managers.

### 2.2 Data Collection

Semi‐structured interviews were undertaken with 32 participants in person or via telephone/video calls over 6 months in 2016. Interviews of approximately one hour focused on the experience of undertaking health promotion within the institutional setting, including perspectives on enablers and strategies for engaging staff. Interviews were transcribed verbatim. Each participant was provided with the research questions (Supplementary File 1) and consent form in advance. Interview questions were developed utilising health promotion [18] and organisational change theory [12, 19] to guide discussion through the policy, programme development and implementation journey (Supplementary File 1). The constant comparative method within grounded theory was used throughout data collection to compare and categorise ideas and ensure data saturation [17].

### 2.3 Data Analysis

Interview transcripts were analysed inductively, and themes were established using NVivo software (version 11). Open coding was employed to examine and understand participants' statements. Three stages of coding were undertaken [20]. The first cycle of inductive coding resulted in descriptive codes generated to group participants' statements; most statements fell into multiple codes. Axial coding was then applied to refine the meaning attributed to participant statements and to refine the categories statements assigned [17]. The final step was selective coding to refine the themes and establish core categories to build a complete story of the findings [17, 20].

### 2.4 Researcher Positionality and Reflexivity

P.T. conducted all interviews and led the data analysis, with the coauthors contributing to the analysis throughout the data collection period and following completion of data collection in 2016–2017. All authors are university‐based academics, two (P.T. and C.M.B.) having had lead roles in the authors' university's move to smoke‐free campuses in 2014. While this provided contextual knowledge of the complex organisational and institutional environment in which health‐promoting policies are developed and implemented, it also introduced the potential for the researchers' professional experiences and assumptions to influence data analysis and interpretation. Researcher bias was identified upfront and mitigated by using the other coauthors as reference points to ensure there was no undue influence [20]. To support reflexivity, analysis was undertaken collaboratively, with interpretations and emerging themes discussed among the research team throughout the analytic process. The analysis was completed in 2017 and has not subsequently been re‐analysed. Accordingly, the findings represent the research team's interpretation of participants' accounts within the policy and institutional context of that period.

## 3 Results

The findings highlight the organisational factors that impacted smoke‐free policy and programme development and implementation in smoke‐free campus programmes launched by 2016. Interviews revealed a range of facilitators for change within the university context; the first was the range of motivations that enabled a shift to a smoke‐free campus, and the second related to the actions needed to implement a smoke‐free campus.

### 3.1 Participant Characteristics

In total, 32 participants were included, 20 males and 12 females, all of whom had completed tertiary‐level education. Most were aged between 50 and 69 years (*n* = 26, 80%). Approximately half (*n* = 18, 56%) were in senior management roles, including the university executive, and approximately half (*n* = 18, 56%) had worked in a university for more than 10 years (Table 1).

**TABLE 1:** Participant characteristics.

Characteristic	*N*
Gender	
Female	12
Male	20
Level of employment	
Senior management (includes executive)	18
Manager of professional staff	5
Facilities and infrastructure staff	3
Academic staff	1
Professional staff	5
Years of university employment	
< 5 years	5
5–10 years	9
> 10 years	18

### 3.2 Motivations for Smoke‐Free Campus Policies

Participants described a range of motivations that enabled campuses to become smoke‐free. Gaining buy‐in from the university executive was critical for smoke‐free campus development. Participants described the importance of identifying motivating factors to encourage change and engage in health promotion activities.

#### 3.2.1 Promoting Health Within the University Community

All participants cited the importance of creating a university environment that is healthy for both staff and students. The consideration of a healthy environment was one of the key motivating factors that led to the establishment of smoke‐free policies that were designed to reduce harm, influence healthy behaviours and normalise smoke‐free lifestyles.

We want a healthy environment. That's our goal—healthy staff and students. (Senior management)

Participants emphasised the importance of ensuring the campus was healthy for everyone, including both smokers and non‐smokers. The result of a smoke‐free campus was twofold. First, it could assist smokers to reduce their own smoking; second, this policy could also reduce second‐hand smoke exposure.

It was about creating a healthy work and study environment. And that meant firstly, removing exposure to second‐hand smoking and secondly, supporting the staff and students who might want to give up smoking. Thirdly, to change the world for those who didn't think they wanted to give up smoking to suddenly make it seem more desirable or possible, and fourthly, to have a cleaner, healthier environment. (Senior management)

An additional consideration was improving the university environment and health of the university community. Participants maintained that it was the role of the university to create a healthy environment.

We have a duty of care and obligation to protect people from the harmful effects of cigarettes. (Senior management)

#### 3.2.2 Institutional Reputation and Sector Leadership

While the health of university communities was a motivating factor for smoke‐free health promotion policy and interventions, so too was establishing a broader healthy institutional culture and the public visibility of this investment. Projecting the public image of a smoke‐free campus was considered important for the university's reputation in maintaining competitiveness in student recruitment and retention, as well as student satisfaction.

We wanted to be seen as the university‐of‐choice because we are healthy and safe and wanted to promote that. (Senior management)

Being considered a sector leader, either nationally or within their university group, was important. For some, being the ‘first’ university in their network or State/Territory to implement smoke‐free policies was a critical point that could be leveraged to influence change.

We wanted to be the first, and that was part of the sell to leadership…. (Senior management)

Of equal importance was the desire for a university not to be last or seen to be lagging other universities. To be out of step with the sector was considered potentially detrimental to university reputation.

The university didn't want to be left behind by the other universities so that was an incentive. (Manager professional)

Participants highlighted the importance of their ‘identity’ when making this change. Participants described their university as a place that should be healthy. This was especially important for universities that delivered health education, research, and services.

Health is a very strong part of the university program … and would have been a driving force in going smoke‐free. (Facilities and infrastructure)

Participants described the possibility of a perceived contradiction if their university was engaged in health promotion research and policy, yet they were not implementing these findings within their own setting. Many participants highlighted the importance of external perceptions that the university was willing to ‘walk the talk’.

Our strongest faculty with the best reputation is our health faculty. If we are running all these programs associated with health but we can't create a healthy campus environment, then it was going to be contradictory. (Manager professional)

#### 3.2.3 Alignment With Societal Norms and Legislation

Reputation and sector leadership were influential in establishing smoke‐free practice. Although these programmes were considered good for university reputation, they were not seen as groundbreaking but more in step with social norms. The final key facilitator for the change to a smoke‐free campus was the perception that the broader community expectations were that public spaces were smoke‐free. This was combined with evolving legal restrictions on smoking in public spaces, providing universities with both a catalyst and justification for change

We're not trailblazers. This just keeps us in step with what society expects. (Senior management)

To be compliant with legislative changes, universities needed to implement and enforce policies such as increasing the distances that smokers had to be from building entry points (windows and doors).

Public policy is changing, no smoking in buildings, no smoking in public places, etcetera; this was obviously having an overall influence. (Senior management)

Legislative requirements provided leverage for change but gave an opportunity for individual universities to consider the breadth of the policy and intervention they planned to implement, for example, providing universities with the discretion on whether to establish dedicated smoking zones on campus. The university values, the existing breadth and scope of health research and impact on university reputation were all influential in determining the types of policies, degree of resource commitment and the implementation strategies employed.

I certainly got a sense from the executive that they were reluctant to do a total ban … so we ended up with the designated areas. (Senior management)

The role of universities in having a positive influence on society was also a key point of discussion for many participants who suggested that smoke‐free campuses would be seen by the broader community as a positive initiative. Participants considered their role as public institutions as one that comes with a responsibility to model health‐promoting behaviours and be aligned with societal values. As educational institutions, participants suggested that the public would expect universities to make this change.

The university likes to view itself as a positive influence on society, and I think they saw this as an opportunity. (Professional staff)

### 3.3 Enablers for Implementation

The second major theme apparent from the interviews was the range of enablers that were considered important when implementing a smoke‐free campus. The motivations and willingness of the senior executive were a stepping stone to implementing the policy change; however, gaining buy‐in from the university community (staff and students) was also important, yet not always straightforward. For implementation to progress, leadership, whole‐of‐community engagement and utilising current services and/or capacity building were required.

#### 3.3.1 Leadership, Champions and Executive Support

Executive leadership, particularly from VCs, was considered by participants to be the most influential internal factor in determining whether a smoke‐free policy advanced from idea to implementation. Participants consistently noted that active and visible VC support expedited policy development, secured cross‐university buy‐in and legitimised the initiative in internal negotiations.

Our VC [Vice Chancellor] was willing to walk the campus and personally speak to smokers. (Senior management)

Participants described the important role that those in senior positions played when championing the smoke‐free policy, enabling those in less senior positions to advance the change. Senior engagement in the campaign for change was seen as commitment across the leadership group.

Having a senior sponsor who was committed to it as a champion was critical. (Manager professional staff)

Engagement from the university executive was valued because it resulted in committed resourcing. Resourcing was in the form of human resources and allocated funding to support initiatives. All participants saw resourcing as fundamental to the success of the initiative, in enabling the university community to adapt and engage with the changes.

These things do take resources, and the problem is our health, wellbeing and safety committees are always under‐resourced, and then you've got to cover an enormous brief, which has a lot of compliance‐based requirements. (Senior management)

An important component of resourcing was the capacity of the university community and the presence of policy champions to support implementation. When senior leadership support was secured, champions among staff and students, using evidence‐based advocacy to strengthen engagement and build capacity, were able to be deployed. Participants identified champions at multiple levels of influence, including roles in student wellbeing, health promotion, occupational health and safety and facilities management. These individuals were pivotal in facilitating internal dialogue, establishing working groups and sustaining momentum throughout the implementation process.

It was our wellbeing officer who kept the pressure on. She had the passion, she had the data, and she brought people together. (Professional manager)

Champions often leveraged internal relationships and reputation to build support across faculties and departments. Where these individuals had credibility and a history of leading change, participants described a more successful policy progression. Champions sometimes engaged with the smoke‐free policy because of a personal experience with smoking.

I am an ex‐smoker, and I come from a time when you used to smoke in the office environment and being an ex‐smoker now, I cannot believe what it was like, what I was like towards colleagues as well, and that's such a selfish approach to being able to smoke where you want when you want… I just have a whole different outlook on smoking, and the idea of a smoke‐free environment is something that I think is a really positive one. (Senior management)

#### 3.3.2 Organisational Culture and Faculty Engagement

The support from leadership and on the ground enabled universities to communicate and implement the smoke‐free policy using a considered and collaborative approach. This approach also enabled a whole‐of‐university attitude and often leveraged academics and faculty to engage through research, design and implementation, to shape operational decisions, ensuring that health promotion initiatives were grounded in evidence. One example involved a health faculty academic serving on a university health and safety committee, where their research knowledge and persuasive advocacy influenced the adoption of a 100% smoke‐free policy (no designated smoking areas).

The academic was able to talk about the research and talk about the opportunity to influence new students at the university… their commitment to go all the way was really good. Otherwise, we might have gone to designated smoke areas because that was the easy option. (Manager professional)

Faculties, especially those with health‐related expertise, offer direct access to student populations, advanced research methodologies, and subject matter expertise, positioning them to play a leading role in shaping evidence‐based, campus‐wide health promotion strategies. Not all universities engaged with their health faculties and considered this as important for health promotion implementation.

We have a faculty of health; I think we could've used the health faculty as greater leverage in this, greater engagement with it. I don't think we did enough… I mean even if it was just within their own faculties, it would have a huge impact because that's where we have the most students, the most staff. (Manager professional)

Participants stated the importance of having all faculties and divisions on board, not only for research and evidence input, but for greater engagement and socialisation of the policy and interventions.

I think the key thing that really stands out for me is the faculty divisional committee connection. We talk a lot in the university now about cross‐unit collaboration, and that was the key I think to make it a success. (Senior management)

#### 3.3.3 Leveraging Existing Resources and University Community Support

Institutional readiness, including the existence of pre‐existing health committees, policy templates, or compliance frameworks, was also associated with implementation. Links between health faculties and wellbeing services were identified as under‐utilised opportunities at some institutions. Alternatively, those that had connections and established collaboration suggested implementation was more efficient.

We already had a healthy campus agenda, so smoke‐free just fit with everything else we were doing. (Health promotion officer)

Few participants articulated the ways that health promotion could or did sit across the university structure or within the university's broader strategy or commitment. There was a recognition of the importance of shifting these values across the university to embed health more broadly.

There isn't that deep‐down commitment that feels that if we invest time, resources, and effort in health and wellbeing, there'll be a payoff with staff satisfaction, or there'll be a payoff with staff retention, or there'll be a payoff somewhere. I don't know if there is that whole business commitment around health promotion. (Manager professional staff)

The institutional culture, including attitudes towards health, perceptions of student and staff rights and traditions of health promotion, played a substantial role in shaping receptivity to smoke‐free initiatives. Universities that positioned themselves as health‐promoting institutions, or that had previously adopted policies such as healthy eating or active transport, were more likely to embed smoke‐free initiatives as part of an overarching wellbeing strategy.

There was really a very broad general support for the policy among staff and students. (Manager professional)

Participants highlighted university community support for smoke‐free campuses, especially among students who had grown up with smoking restrictions. However, a minority of staff and student smokers raised human rights objections, particularly in institutions that adopted a more top‐down implementation approach.

A few thought it was about individual rights, but they were the vocal minority. (Manager, professional)

Concerns about compliance and enforcement were common, especially on large campuses. Smoke‐free policies provided a long‐term commitment, and a whole‐of‐university approach supported the community through the change.

## 4 Discussion

This study explored the organisational factors that influenced the development and implementation of health promoting policy in Australian universities undertaking a transition to smoke‐free campuses by 2016. Findings from this study indicate that internal organisational factors, executive leadership, institutional culture and the presence of internal champions are all integral to health promotion policy development and implementation.

Institutions that were prepared to engage with change allocated greater resources and achieved broader engagement across the university community, whereas less ready institutions experienced fragmented support and limited follow‐through. Institutional culture was central to this readiness, influencing receptivity to smoke‐free adoption and reinforcing Lewin's [19] assertion that sustainable change requires embedding new practices within organisational norms and values. Universities with pre‐existing commitments to wellbeing, particularly those with health and wellbeing strategies, had established conditions in which smoke‐free policies were perceived as congruent with institutional values, reflecting the healthy settings approach's emphasis on cultural integration rather than isolated interventions [13]. Reputation further intersected with culture, as external perceptions of universities influenced internal policy priorities and the extent to which health promotion aligned with institutional identity and strategy [21, 22]. However, in institutional cultures that strongly value autonomy and individual choice, health promotion initiatives risk being framed as paternalistic, echoing Crawford's [23] critique of ‘healthism’. This tension highlights the importance of systems‐based approaches that situate health policies within shared organisational goals, particularly given evidence that policies introduced without such alignment often result in limited implementation and minimal behaviour change in practice [9, 24].

Linking smoke‐free policy to a duty of care and occupational health and safety (OH&S) obligations increased senior‐level buy‐in. Health professionals within academia and university health services could advocate for sustained health and wellbeing policy and practice, utilising academic links to showcase associations between healthy environments and student retention, academic attainment and staff productivity [25]. By creating a ‘sense of urgency’ and tapping into risk management frameworks familiar to senior leaders, the issue may be further prioritised [12]. The obligation to change was a strong driver for engaging executive leadership and building committees or working groups across university faculties and divisions, which supported implementation.

Beyond organisational obligation, smoke‐free policy provided an opportunity for a whole‐of‐university approach, combining top‐down leadership with bottom‐up engagement [26]. Internal champions acted as change agents, translating health promotion goals into practice [27]. By bridging academic and operational domains, champions distributed leadership, fostered collaboration across traditionally siloed university structures and sustained implementation [12]. Implementation also drew largely on existing institutional resources, including campus health services, policies and wellbeing strategies, which provided important infrastructure for embedding health‐promoting initiatives.

For health promotion to become embedded in university systems, leaders need to adopt a dual‐framework approach that integrates health promotion [28] with organisational change theory [12]. Aligning change with the vision of the Okanagan Charter [3], the actions within the Limerick Framework [5] and the Healthy Settings approach [13] helps define university readiness, the vision, principles and participatory processes. By intentionally aligning health promotion practice with organisational change processes, universities can transition from ad‐hoc health and wellbeing intervention programmes to a systemic, evidence‐based culture of health, reinforcing their dual role as agents and settings of transformative change and as community leaders in health promotion.

## 5 Strengths and Limitations

A limitation is the interval between data collection and publication. Although the data were analysed in 2016–2017, the manuscript has been prepared following recent changes in health promotion and wellbeing expectations placed on Australian universities in the 2025 Australian Universities Accord. There have been substantial changes in tobacco control and the broader higher education context over the nine intervening years. This may limit direct translation from this case study to the current university policy environment. However, the concurrent nature of the interviews is also a key strength, as participants provided accounts during the active development and implementation of smoke‐free campus policies, rather than retrospectively recalling events a decade later.

## 6 Conclusion

This study contributes contemporaneous insights into the organisational and contextual factors that enabled smoke‐free policy change in Australian universities during a formative period of tobacco control. By examining accounts from institutional leaders and other university staff involved in policy development and implementation, insights into how organisational change was negotiated within the complex university setting were gained. The findings demonstrate that executive leadership, internal champions, organisational resources and wider institutional priorities interacted to support policy development and implementation. Executive endorsement provided legitimacy and authority for change, while internal champions helped translate policy intent into practice and sustain implementation.

In university settings, there is a need to consider the policy and organisational context that makes healthy options the easiest choice for staff and students alike. This requires advocacy for change, mediating people, strategies and policies and enabling change through research and resources. Embedding policy where the intent is reflected in organisational strategic plans, senior management KPIs, committee agendas and so forth creates the supportive environment needed for health promotion. These findings are situated within the tobacco‐control and higher education context of 2016 and do not reflect the substantial expansion of smoke‐free and vaping regulation since that time. However, these case studies do provide a historically grounded account of how universities navigated policy change during this formative period and illustrate organisational processes relevant to emerging health priorities in complex academic institutions. Applying organisational change theory can help situate such initiatives within broader, coherent strategies for health‐promoting organisational change, rather than treating them as isolated interventions.

## Funding

The authors have nothing to report.

## Ethics Statement

Ethics approval was granted by Deakin University Human Research Ethics Committee (approval number HEAG‐H 101_2013).

## Conflicts of Interest

There authors declare no conflicts of interest.

## Supporting information


**Data S1:** Supplementary File 1.

## Data Availability

The data that support the findings of this study are available from the corresponding author upon reasonable request.

## References

[1] Department of Education and Training , Higher Education Standards Framework (Threshold Standards) 2015 (Australian Government, 2015), https://www.legislation.gov.au/Details/F2015L01639.

[2] M. Dooris , “The “Health Promoting University”: A Critical Exploration of Theory and Practice,” Health Education 101 (2001): 51–60, 10.1108/09654280110384108.

[3] International Conference on Health Promoting Universities and Colleges , Okanagan Charter: An International Charter for Health Promoting Universities and Colleges (University of British Columbia, 2015), https://open.library.ubc.ca/cIRcle/collections/53926/items/1.0132754.

[4] P. Taylor , R. Kennedy , M. Dolf , C. Woods , A. Tierney , and C. Norton , “Health Promoting Campuses: A Time for Global Reflection and Renewed Action,” Health Promotion International 40, no. 3 (2025), 10.1093/heapro/daaf086. 40516117

[5] International Health Promoting Campuses Network, University of Limerick , The Limerick Framework for Action: Advancing the Global Health Promoting Campuses Agenda (University of Limerick, 2025).

[6] Australian Government, Department of Education , Australian Universities Accord (Australian Government, 2024), https://www.education.gov.au/australian‐universities‐accord/resources/final‐report.

[7] M. Hutchesson , M. Whatnall , S. Hunt , et al., “Implementation of Health and Wellbeing Policies and Programs for Students: Views of Australian Universities,” Health Promotion Journal of Australia 35, no. 3 (2024): 840–843, 10.1002/hpja.808. 37705141

[8] L. Sanci , I. Williams , M. Russell , et al., “Towards a Health Promoting University: Descriptive Findings on Health, Wellbeing and Academic Performance Amongst University Students in Australia,” BMC Public Health 22 (2022): 2430, 10.1186/s12889-022-14690-9. PMC9792939 36575509

[9] X. Luu , “Towards Comprehensive Mental Health Promotion in Australian Universities: A Call to Action With Priorities for Institutional Change,” Health Promotion Journal of Australia 36, no. 1 (2025): 1–4, 10.1002/hpja.891. PMC11730811 38866387

[10] C. Sáenz , E. Boldú , and J. Canal , “Health Promoting Universities: A Scoping Review,” Health Promotion Journal of Australia 36, no. 2 (2025): e939, 10.1002/hpja.939. 39912192

[11] Department of Health , National Tobacco Strategy 2012–2018, by Intergovernmental Committee on Drugs (Commonwealth of Australia, 2013).

[12] J. P. Kotter , Leading Change (Harvard Business Review Press, 1996).

[13] M. Dooris , G. Dowding , J. Thompson , and C. Wynne , “The Settings‐Based Approach to Health Promotion,” in Health Promoting Universities: Concept, Experience and a Framework for Action, ed. A. D. Tsouros , G. Dowding , J. Thompson , and M. Dooris (WHO Regional Office for Europe, 1998), 21–32.

[14] B. Poland , G. Krupa , and D. McCall , “Settings for Health Promotion: An Analytic Framework to Guide Intervention Design and Implementation,” Health Promotion Practice 10 (2009): 505–516, 10.1177/1524839909341025. 19809004

[15] World Health Organization , Healthy Settings (WHO, 2017), http://www.who.int/healthy_settings/en/.

[16] P. Taylor , A. Allisey , F. H. McKay , C. Stevenson , and C. M. Bennett , “Health Promotion Policy in Australian Universities—A Smoke‐Free Policies Case Study,” International Journal of Health Promotion and Education (2023): 1–15, 10.1080/14635240.2023.2270978.

[17] J. M. Corbin and A. L. Strauss , Basics of Qualitative Research: Techniques and Procedures for Developing Grounded Theory, 3rd ed. (Sage Publications, 2008).

[18] B. D. Poland , L. W. Green , and I. Rootman , Settings for Health Promotion: Linking Theory and Practice (Sage Publications, 2000).

[19] K. Lewin , Resolving Social Conflicts: Field Theory in Social Science (American Psychological Association, 1997).

[20] M. B. Miles , A. M. Huberman , and J. Saldaña , Qualitative Data Analysis: A Methods Sourcebook, 3rd ed. (Sage Publications, 2014).

[21] C. Free and M. Garbuio , “Is There Any Strategy in University Strategic Plans? A Comparative Study of the Tone and Content of Strategic Plans of Australian Universities,” Australian Journal of Management 51, no. 3 (2025): 675–705, 10.1177/03128962251350347.

[22] K. Nielsen and A. Noblet , Organizational Interventions for Health and Well‐Being: A Handbook for Evidence‐Based Practice (Routledge, 2018).

[23] R. Crawford , “Health as a Meaningful Social Practice,” Health 10, no. 4 (2006): 401–420, 10.1177/1363459306067310. 16973678

[24] M. Hutchesson , T. Burrows , L. Couper , et al., “Policies and Strategies to Promote Optimal Mental Health of Australian University Students: A Document Review,” Health Promotion Journal of Australia 36, no. 3 (2025): e70066, 10.1002/hpja.70066. PMC12202985 40574310

[25] M. Dooris , S. Doherty , and J. Orme , “The Application of Salutogenesis in Universities,” in The Handbook of Salutogenesis, ed. M. B. Mittelmark , S. Sagy , M. Eriksson , et al. (Springer, 2017), 237–245. 28590663

[26] C. MacDougall and Y. Laforet‐Fliesser , “The Evolution of Healthy Schools in Ontario, Canada: Top‐Down and Bottom‐Up,” in Case Studies in Global School Health Promotion: From Research to Practice, ed. C. Aldinger and C. V. Whitman (Springer, 2009).

[27] D. Nutbeam , “Health Education and Health Promotion Revisited,” Health Education Journal 78, no. 6 (2019): 705–709.

[28] World Health Organization , Ottawa Charter for Health Promotion (WHO, 1986).

