# Supplementary material for: The Organisational Drivers That Enabled Health Promoting Policy Change Within Australian Universities—A 2016 Move to Smoke‐Free Campuses Case Study
Source: Health Promot J Austr. 2026 Oct 20;38(1):e70254. doi: 10.1002/hpja.70254 (PMC13682199; doi:10.1002/hpja.70254)
Supplement: Supplementary file 1 [file HPJA-38-0-s001.docx]

Supplementary File One

Interview Guide

Research Aim:

To understand the facilitators and barriers to developing and engaging staff and students in the implementation of smoke-free (SF) policy & programs in the Australian university setting.

## Objectives:

- To gather data on the internal or external factors that motivated universities in Australia to move to campuses with smoking restrictions.
- To understand the implications of developing smoke-free policy or programs within Australian universities.
- To understand community engagement throughout the development and initial implementation phase of the smoke-free policy or program.

## Interview Scope:

These interviews are the second phase of data collection. The first phase was a policy analysis of all Australian universities’ smoke-free (SF) policies and documents. By undertaking interviews with key stakeholders involved in smoke-free campus development we aim to gain in-depth understanding of the motivations for universities in Australia to move beyond their legislative requirements in regards to smoking on campus. As well as understand the barriers and facilitators to undertaking this shift.

Each participant will be asked some general questions prior to the interview. These are also to clarify particular details and events.

This interview guide is flexible and will be phrased to suit the participant’s knowledge, experience and conversation direction, based on the feedback from the demographic questionnaire or opening questions.

## Clarifying questions:

### Name

### DOB

### Gender

### Qualifications – what are your qualifications to date?

### What is your current position title (if this has changed since you worked on smoke-free, can you please list all your titles)?

### How many years have you worked in this current role at this University? If you were in a different role whilst undertaking SF – how long were you in that role?

### What are your key responsibilities in the role at the time you were involved in SF?

## Developing a smoke-free focus within the University:

Opening statement: I’d like you to think about the smoke-free program that has been implemented at your university.

**I am most interested in your perspectives on what influenced the smoke-free initiative in your university.*

1. **Have you participated in your university’s SF program?**
2. **How do you refer to the smoking restrictions on campus, what do you call it or how do you refer to it?**
3. **When was the SF initiative introduced?**
4. **Can you give me an example of your participation? Can you give me an example of your participation? What was your involvement in the development phase of smoke-free, pre implementation?**
5. **Why do you believe the initiative was introduced?**
6. **From your perspective, who led the introduction of SF across your University?**
7. **What were the processes involved in the development?**
8. **What are your thoughts in relation to what went well and what could have been improved?**
9. **Do you think the timing of the introduction of smoking restrictions was because of anything specifically?**
10. **How long was the period of planning before implementation?**
11. **Why do you think it took this amount of time?**
12. **What were the approval processes involved in smoke-free sign off?**
13. **Who was involved in the development of the SF initiative?**
14. **Can you tell me a little about the work that you undertook in the development of the policy/program?**

** Thinking about the smoke-free initiative you’ve described, let’s now focus on the implementation and particularly engaging staff and students.*

15. **Were you involved in the implementation of the move to SF campuses?**
16. **Can you tell me about the decision making process for implementation?**
17. **Can you tell me about the consultation process around implementation?**
18. **Can you tell me how you went about engaging staff and students?**
19. **How was the communication developed/managed across the University?**
20. **Was this process monitored? If not, why? If yes by whom, when and how?**
21. **What successes and challenges can you describe about the engagement phase?**
22. **What was the decision-making process for this project?**
23. **Are you aware of other universities in your State/Territory or nationally that have moved to SF?**
24. **How do you think health promoting policy sits across the University’s strategic focus?**
25. **In summary, why do you believe your University was motivated to implement SF across your campuses? What would you say are the top three motivations for your university to move beyond the legislative smoke-free requirements?**
26. **If someone was to step into your shoes and implement SF at your university, what would be your top points for them to succeed?**

I will be speaking with a range of people from your university. Would you recommend I speak with anyone else in particular?

Is there anything else you would like to say?

Thank you.
